# Effectiveness of Cognitive Rehabilitation on Cognition and Quality of Life in Chemotherapy-Induced Cognitive Impairment: Protocol for a Randomized Control Trial

**DOI:** 10.2196/66011

**Published:** 2025-11-20

**Authors:** Gayatri Surendra Kaple, Moh’d Irshad Qureshi, Raghumahanti Raghuveer, Sharath Hullumani

**Affiliations:** 1 Faculty of Neuro Physiotherapy Datta Meghe Institute of Higher Education and Research Wardha India; 2 Faculty of Paediatric and Neonatal Physiotherapy Datta Meghe Institute of Higher Education and Research Wardha India

**Keywords:** Cognitive Rehabilitation, Cancer, Chemotherapy, Physiotherapy, Cognitive Impairment, Chemotherapy-induced cognitive impairment, cognitive, cognition, cognitive impairments, cognitive decline, Effectiveness, Cognitive Rehabilitation, Quality of Life, QoL, patient, patients

## Abstract

**Background:**

Abnormal cell growth that can multiply and transfer to other bodily parts is referred to as cancer. Chemotherapy is a standard treatment for cancer patients. During or after treatment, patients may have varying degrees of cognitive impairment caused by the drug, which can negatively impact their quality of life. Cognitive impairment brought on by chemotherapy is one of the most frequent side effects that occur both during and after chemotherapy. Cognitive effects after and during chemotherapy have not been addressed in patients.

**Objective:**

To evaluate the effectiveness of cognitive rehabilitation on cognition and quality of life in chemotherapy-induced cognitive impairment.

**Methods:**

In this Randomized Control Trial (RCT), 60 subjects with chemotherapy-induced cognitive impairments secondary to any cancer will be randomised into 2 groups by the sequentially numbered opaque sealed envelope approach. The cognitive functions will be measured as the outcome measures, which include all the recall strategies, executive function tasks, information processing speed, and attention. Both groups will receive intervention every day for 4 weeks. Wilcoxon signed the rank test for data analysis, and students paired the “t” test, which will be used to study within-group effects. Mann-Whitney U Test and students Unpaired “t” Test will be used to compare groups at pre-treatment and post-treatment.

**Results:**

As of November 2024, we will enrol 60 participants, and the data collection process will start from November 2024- November 2025. The expected results of the effect of cognitive rehabilitation on cognition and quality of life in chemotherapy-induced cognitive impairment in cancer patients will be significant, and the expected results will be published at the end week of November 2025.

**Conclusions:**

The study design is a framework to plan and test the feasibility of the intervention. The cognitive therapy will improve the cognition in chemotherapy-induced cognitive impairment patients.

**Trial Registration:**

Clinical Trials Registry India CTRI/2024/04/065805; https://ctri.nic.in/Clinicaltrials/pmaindet2.php?EncHid=MTAyNTE1&Enc=&userName=

**International Registered Report Identifier (IRRID):**

DERR1-10.2196/66011

## Introduction

### Background

Cancer is the leading cause of death and a major limitation to increasing life expectancy in every nation across the globe. According to the World Health Organization (2019) [1], in 112 out of 183 countries, cancer was a primary cause of mortality before the age of 70, and it ranked third or fourth in 23 additional countries. While death rates from conditions such as stroke and coronary heart disease are declining, cancers have risen in prominence as a leading cause of mortality [2]. Patients with noncentral nervous system tumors often experience cognitive symptoms, commonly referred to as “cancer-related cognitive impairment” (CRCI). This condition, primarily studied following chemotherapy, involves deficits in working and short-term memory, attention, executive function, and processing speed. After chemotherapy, up to 75% of patients with cancer report cognitive problems, and approximately 35% continue to experience cognitive dysfunction for 5-10 years after completing treatment [3].

As a result of population growth and aging, as well as shifts in the distribution and prevalence of major cancer risk factors, the global burden of cancer incidence and mortality is continuously increasing. An estimated 58.3% of cancer deaths in both sexes and half of all cancer diagnoses are expected to occur in Asia, which is home to 59.5% of the world’s population. According to GLOBOCAN 2020 estimates, India is projected to record 2 million new cancer cases by 2040, a 57.5% increase from 2020 [2]. Chemotherapeutic drugs have significantly improved cancer survival rates since their discovery and development over the past several decades. The 5-year survival rate across various cancers is around 60%, with skin melanoma, breast cancer (BC), and prostate cancer showing the highest rates. However, all treatments have side effects. Chemobrain, chemofog, or chemotherapy-induced cognitive impairment (CICI) is among the most common adverse effects of chemotherapy [4].

CICI is primarily investigated through neuroimaging studies of the brain, which reveal decreased gray matter density in the frontal, temporal, cerebellar, and right thalamic regions [5]. The prefrontal cortex and hippocampus are the primary centers of learning and memory; therefore, any damage to these areas results in cognitive dysfunction [6]. Consequently, altered behavior and memory are associated with impaired neuronal functioning in the hippocampus and frontal cortex [7]. Moreover, changes in cerebral white matter, mitochondrial dysfunction, and depletion of neural progenitor cells in the dentate gyrus contribute to cognitive impairments [8]. However, limited information is available regarding the precise mechanisms involved. Triple therapy with methotrexate, doxorubicin, and cyclophosphamide has been shown to increase peripheral and central levels of proinflammatory cytokines while reducing anti-inflammatory cytokine levels. This imbalance in the cytokine system disrupts neural plasticity and impairs cognitive function. Neuroimaging studies have shown reduced dendritic spine development in this model [9]. In summary, chemotherapy negatively affects cognition. Current reports on BC survivors highlight the occurrence of CICI. Variations in cytokine concentrations and the degree of cognitive impairment are associated with different chemotherapy regimens and dosages. However, the onset of cognitive disturbances remains unclear. The cellular targets, chemical composition of the drugs, dosing schedule, and numerous other confounding factors all play significant roles in determining the central nervous system’s inflammatory or neurotoxic side effects. Peripheral inflammation may also contribute to cognitive problems. Therefore, it is essential to closely monitor the effects of each specific drug in both preclinical and clinical studies [10].

Methotrexate is one of the chemotherapeutic agents known to cause vascular damage through its antiangiogenic activity [11]. Overall, the complex nature of anticancer therapy and its multiple mechanisms of action may trigger multifaceted symptoms of cognitive disorders and behavioral toxicities. In both clinical and experimental settings, doxorubicin is commonly administered to patients with BC who subsequently exhibit cognitive impairment, structural abnormalities, and brain mitochondrial dysfunction [12]. Platinum-based agents (such as cisplatin, oxaliplatin, and carboplatin) disrupt protein synthesis by cross-linking mitochondrial DNA, which is more susceptible to irreversible mutation than nuclear DNA [13]. Studies in mouse models have shown that deep brain penetration and inhibition of neural stem cell proliferation underlie cisplatin-induced cognitive impairment [14].

Crucial regions of the cerebral cortex and peripheral neurons undergo structural degradation due to taxanes (paclitaxel or docetaxel). Studies in neuropathic rodent models have demonstrated that mitochondria are particularly susceptible to damage induced by taxanes [8]. Administration of the same drug combination for 4 weeks led to increased expression of interleukin-1β and tumor necrosis factor-alpha in the rat corpus callosum, while interleukin-10 levels were reduced, resulting in impaired working memory function [15].

In summary, chemotherapy adversely affects cognitive function. Current reports on BC survivors highlight the occurrence of CICI. Variations in drug dosage and cytokine concentration levels across different chemotherapy regimens are associated with differing degrees of cognitive impairment [16]. Research into the mechanisms underlying the development of these cognitive disorders is still ongoing. The deleterious effects of inflammation or neurotoxicity on the central nervous system largely depend on cellular targets, drug composition, dosage schedules, and other confounding factors. Cognitive disturbances may also arise from peripheral inflammation. Therefore, it is essential to closely monitor the effects of each specific drug in both preclinical and clinical studies [17].

Conventional treatment approaches include aerobic exercise and cognitive behavioral therapy. These involve activities such as brisk walking, jogging, cycling, and engaging in cognitive skill exercises or workbooks. Studies have shown that, in addition to medical treatment, combining cognitive and aerobic exercises helps reduce symptoms of cognitive impairment without causing complications. Therefore, such interventions should be recommended for all patients with cancer experiencing CICI.

### Objectives

- To determine the effect of cognitive rehabilitation on improving cognition in patients with CICI across all cancer types.
- To compare the effectiveness of cognitive rehabilitation with conventional therapy in patients with CICI.
- To determine the effect of cognitive rehabilitation on improving the quality of life in patients with CICI across all cancer types.

## Methods

### Trial Design

This is an equal allocation, parallel-group, 2-arm superiority study, with the sample size distributed equally between the 2 groups. Patients will be evaluated according to predetermined inclusion and exclusion criteria and will be recruited from Siddharth Gupta Memorial Cancer Hospital in Sawangi, which is affiliated with Acharya Vinoba Bhave Rural Hospital. After obtaining informed consent, eligible participants will be selected through simple random sampling using computer-generated random numbers. All patients will be informed about the study procedures and asked to provide written consent before participation. The trial will include patients with a medical diagnosis of cancer who are currently undergoing chemotherapy. Sequence generation will be performed using computer-generated random numbers.

The allocation concealment mechanism will involve simple random sampling. Study participants will be randomly assigned using computer-generated random numbers. Randomization will be conducted by a researcher not involved in participant recruitment. To ensure allocation concealment, opaque sealed envelopes will be used, which will be opened only after participants are deemed eligible for treatment by the research team. Data collection will be carried out after participants are assigned to their respective intervention groups, with outcomes assessed and recorded during the preintervention phase. Postintervention data collection will be conducted after 4 weeks of intervention.

**Figure 1:**
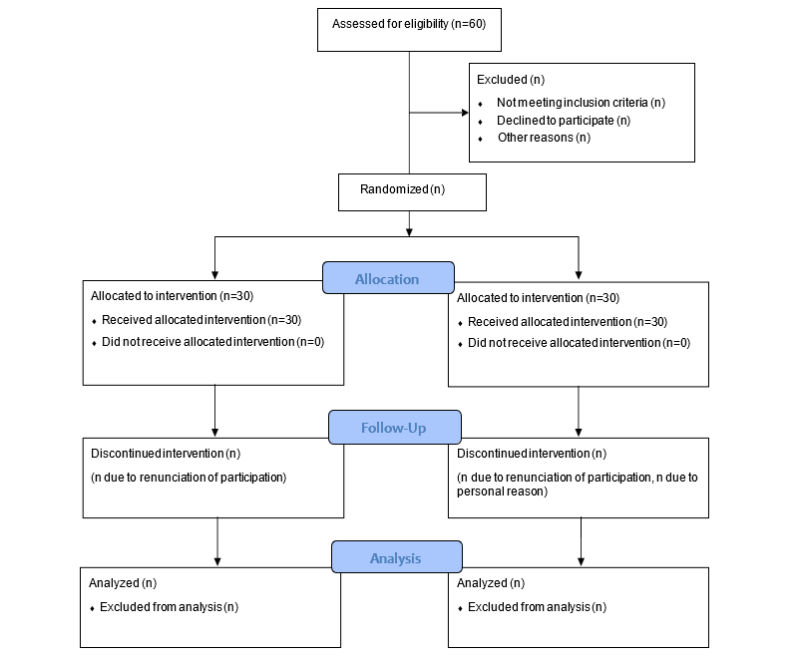
CONSORT (Consolidated Standards of Reporting Trials) flow diagram.

In data management, information obtained will be summarized using frequency and percentage for qualitative data, and mean and SD for quantitative data. Figure 1 presents the CONSORT (Consolidated Standards of Reporting Trials) flow diagram outlining the study design. See Multimedia Appendix 1 for the SPIRIT (Standard Protocol Items: Recommendations for Interventional Trials) checklist.

Descriptive statistics will be used to analyze demographic variables such as age and gender. Measures including mean, SD, frequency (n, %), chi-square test, and independent *t* test will be applied to confirm the homogeneity of descriptive statistics. Inferential statistics between the 2 groups will be assessed using an unpaired (2-tailed) *t* test. All statistical analyses will be performed using SPSS software, version 21.0 (IBM Corp). For this study, a *P* value of less than .05 will be considered statistically significant.

### Ethics Approval

The study was approved by the Datta Meghe Institute of Higher Education and Research Institutional Ethics Committee (approval number DMIHER [DU]/IEC/2024/169; approval date: January 31, 2024). All participants will be informed about the study and will provide written informed consent and assent before enrollment. All data on research participants will be kept strictly confidential. Patient information will be used only with the explicit permission of the individuals. There are no financial or conflicting interests to declare. The principal investigator will be responsible for preserving and maintaining all data collected during and after the study. The final trial dataset will be made available to the principal investigator upon formal request for research and publication purposes only, and access will be granted in a deidentified format.

In accordance with the policies of Ravi Nair Physiotherapy College and Datta Meghe Institute of Higher Education and Research, appropriate care will be provided to research participants if their involvement in the trial results in any harm. All information collected during or after the study will be used solely for academic and research purposes, with the ultimate aim of publication in a reputable journal. The key trial procedures, including enrollment, interventions, and assessments, are summarized in Table 1.

**Table 1:** Demographics and clinical characteristics of the patients.

Confounders		Enrollment	Completed the treatment	Dropout (lost to follow-up/death)
**Age (years)**				
	14-21	✓	✓	✓
	22-30	✓	✓	✓
	31-38	✓	✓	✓
	39-46	✓	✓	✓
	47-55	✓	✓	✓
	56-64	✓	✓	✓
	65-70	✓	✓	✓
**Sex**				
	Male	✓	✓	✓
	Female	✓	✓	✓
**Cancer type**				
	Skin	✓	✓	✓
	Lungs	✓	✓	✓
	Female breast	✓	✓	✓
	Prostrate	✓	✓	✓
	Colon and rectum	✓	✓	✓
	Cervix and uterus	✓	✓	✓
	Head and neck	✓	✓	✓
**Chemotherapy type**				
	Cisplatin	✓	✓	✓
	Paclitaxel	✓	✓	✓
	5-Fluorouracil	✓	✓	✓
	Carboplatin	✓	✓	✓
	Dacarbazine	✓	✓	✓
	Temozolomide	✓	✓	✓
	Nab-paclitaxel	✓	✓	✓
	Oxaliplatin	✓	✓	✓

### Eligibility Criteria

#### Inclusion Criteria

- Recently diagnosed patients with stage I to III cancers who are scheduled to receive a defined number of chemotherapy cycles involving platinum-based compounds.
- Patients currently undergoing chemotherapy treatment.
- Patients receiving taxane- or platinum-based compounds, including cisplatin, carboplatin, or oxaliplatin.
- Patients with grade I or II cognitive impairment as per the National Cancer Institute’s standard toxicity criteria.
- Patients scheduled to receive a minimum of 4 cycles of chemotherapy with any of the aforementioned platinum-based agents.
- Male and female patients aged between 14 and 70 years.
- Patients receiving chemotherapy drugs approved by the National Cancer Board.

#### Exclusion Criteria

- Patients with preexisting cognitive impairment.
- Patients with a history of traumatic brain injury.
- Patients with any prior psychological disorder that could affect cognitive function.
- Patients who have not received any chemotherapy cycles following cancer diagnosis or treatment.
- Patients with a previous history of cancer or prior exposure to chemotherapeutic drugs.
- Patients with neurological conditions affecting the brain or spinal cord, including vascular dementia, multiple sclerosis, amyotrophic lateral sclerosis, Alzheimer disease, Lewy body disease, stroke (cerebrovascular accident), or Huntington disease; as well as those taking medications known to impair cognition, such as benzodiazepines.
- Patients using cholinesterase inhibitors or antipsychotics; those with current or recent (within the past 6 months) alcohol or drug use disorders; those exhibiting behavioral changes such as depression, anxiety, or stress; and individuals with systemic conditions that may interfere with cognition, such as hypothyroidism, folic acid or vitamin B12 deficiency, or viral infections (eg, syphilis, HIV). Patients with visual or speech impairments (eg, aphasia) will also be excluded.

### Selection of Participants

After obtaining ethical clearance from the Institutional Ethics Committee, patients diagnosed with cancer and scheduled to undergo chemotherapy will be identified at Siddharth Gupta Memorial Cancer Hospital. Permission will be sought from the treating residents, and eligible patients who meet the inclusion criteria will be enrolled after obtaining informed consent. Treatment will commence within 1 week of initiating the chemotherapeutic agent, and baseline data will be recorded. Participants will receive cognitive rehabilitation focusing on memory, attention, information processing, and executive function, with the aim of enhancing cognitive performance and improving quality of life. The first outcome measure, the Mini-Mental State Examination (MMSE), will be administered before the initiation of chemotherapy along with baseline data to assess cognitive function. Subsequently, the Montreal Cognitive Assessment will be conducted over 4 chemotherapy cycles. Participants will undergo the intervention 5 days per week, and following the completion of the 4 chemotherapy cycles, postintervention baseline data will be recorded. The outcome measures will be used to assess the degree of cognitive impairment. Follow-up assessments will be conducted after each chemotherapy cycle and weekly after completion of the intervention. Baseline outcome measures will be recorded after 4 chemotherapy cycles, and follow-up evaluations will be carried out once a week for 4 consecutive weeks.

### Participant Timeline

Pre- and postbaseline data will be collected, followed by a 4-week intervention period. After the completion of the 4 weeks, postintervention outcomes will be assessed. The study design is illustrated in the CONSORT flowchart presented in Figure 1.

### Control Group

Participants will perform aerobic exercises and cognitive behavioral training daily. The program will include brisk walking, cycling, jogging, and cognitive skill activities for 45 minutes per day, 5 days a week, over 4 weeks. These activities will not require direct supervision.

### Intervention Group

Domain-specific cognitive exercises to be administered to patients are outlined in Table 2.

**Table 2:** Intervention group.

Problem identified		Physiotherapy intervention	Dosage		
**Memory recall**					
	Mnemonics (face-name recall)	Give a photograph of a relative. Generate a mnemonic after the discussion that will be used to assist recall. During the training phase, using the forward cueing method, increase the cues until the patient identifies the picture.	Run 1 training trial, then test immediately, and again after 30 seconds, 1 minute, 2 minutes, 5 minutes, and 10 minutes.		
	Cueing (number recall)	Generate mnemonics to remember phone numbers. During the training phase, prompt a line in increasing or decreasing order.	N/A^a^		
	Chunking (story recall)	Read a story in front of the patient. Break the story into parts and try to imagine the important key points, such as what, where, who, when, and why.	N/A		
	Method of loci (procedural memory and fluency training)	Prompting and fading, action-based encoding, chaining, and fluency training (names and numbers).	N/A		
	Spaced retrieval (semantic impairments)	Repeated rehearsal of word-picture pairing will improve the ability to name trained items for 4 people. For word association, the therapist will give a word and ask the patient to identify a related word with the same meaning.	N/A		
**Executive function**					
	Naming task	Ask the patient to say the name of any item. Show the item for 5 seconds, then repeat the task for 60 trials.	There were 60 trials in total, and the task lasted 5 minutes.		
	Calculation task	Ask the patient to add or subtract 2-digit numbers. The patient must respond within 5 seconds.	N/A		
	Stroop color-word task	The therapist shows words in different colored fonts. At times, the color matches the word itself, and at other times, it does not. The patient must name the color of the font.	N/A		
	Working memory task	Show the patient a series of numbers for 2 seconds. The patient should then repeat the series.	N/A		
**Attention**					
	Sustained and selective attention	Elemental training: focus on breathing and body-part awareness.	20 minutes of training		
	Alternating and divided attention	Functional training: make a grocery list to prepare meals, select the correct food pictures showing their expiry dates, and determine locations on the map.	N/A		
	Information processing	The perceive, recall, plan, and perform system [18]	N/A		
	Perceive	The patient decides which sensory information to attend to, then analyzes the characteristics of objects, surfaces, and body parts.	10 minutes of training		
	Recall	The patient will recall prior knowledge of the procedure and then apply the relevant knowledge required to perform the task.	N/A		
	Plan	The patient will think, prepare to perform, and make decisions to achieve the goal.	N/A		
	Perform	The therapist will analyze whether the patient is able to initiate and terminate the response, and to continue and persist as the task requires.	N/A		
**Physical fitness**					
	Aerobic exercises [18] (frequency, intensity, interval, and time principle)	Brisk walking, jogging, and static cycling	Frequency: 5-6 times per week; intensity: easy to moderate, approximately 60%-75% of maximum heart rate.		

^a^N/A: not applicable.

### Rationale

The rationale for including cognitive behavioral training in the control group intervention is based on its established effectiveness in addressing cognitive and behavioral disturbances, as demonstrated in several recent studies. Substantial evidence indicates that cognitive behavioral training significantly alleviates anxiety and depression among individuals diagnosed with cancer. Furthermore, a few studies have explored the role of cognitive behavioral training in reducing cancer-related fatigue. Consequently, cognitive behavioral training may serve as a promising approach to mitigating the adverse effects of chemotherapy [19]. A systematic review and meta-analysis further concluded that cognitive behavioral training, when combined with resistance and aerobic exercise, is more effective, as active exercise enhances physical capacity. In addition, cognitive behavioral training contributes to fatigue reduction by improving coping mechanisms for stress and promoting better activity management [20].

By targeting key domains such as attention, memory, processing speed, and executive function, neurocognitive exercises have been employed in an experimental study by Sundar et al [21] to aid cancer survivors in recovering from the cognitive effects of chemotherapy. Compared with aerobic exercises, neurocognitive training was found to produce significantly greater improvements in both cognitive function and overall quality of life. The study further demonstrated that enhancements in cognitive performance among postchemotherapy cancer survivors are strongly correlated with domain-specific cognitive training [21].

### Outcome Measures

#### Primary Outcomes

##### Mini-Mental State Examination

The MMSE comprises 11 questions designed to assess cognitive performance across 5 domains: orientation, registration, attention and calculation, recall, and language. Developed in 1975, the MMSE has a maximum score of 30 points. A total score of 23 or below indicates cognitive impairment, whereas a score of 24 or above is considered within the normal range.

##### Montreal Cognitive Assessment

According to clinicians, the MMSE takes approximately 10 minutes to administer. It assesses various domains of cognition, including visuospatial skills, executive functions, attention, concentration, calculation, language, abstraction, memory, and orientation.

##### Functional Assessment of Cancer Therapy—Cognitive

The Functional Assessment of Cancer Therapy—Cognitive Function version 3 is a 27-item self-report questionnaire designed to assess cognitive concerns among patients with cancer. It comprises 4 subscales, including Physical well-being, Social/family well-being, emotional well-being, and Functional well-being.

#### Secondary Outcomes

##### European Organisation for Research and Treatment of Cancer Quality of Life Questionnaire Core 30

The European Organisation for Research and Treatment of Cancer Quality of Life Questionnaire (EORTC QLQ-C30, version 3) is a 30-item instrument specifically developed to assess health-related quality of life in patients with cancer.

##### Patient-Specific Functional Scale

The Patient-Specific Functional Scale is a self-reported, patient-centered tool used to evaluate functional changes over time. Developed by Stratford and colleagues [22], the scale serves as a versatile measure of function that can be applied to individuals with varying levels of independence.

#### Sample Size Calculation

Calculation of sample size by Cohen effect size by comparing 2 means:

Effect size (d) = (μ_2_ – μ_1_)/σ = 0.8 (estimated)

Considering a large effect size difference of 0.8, the sample size is calculated as follows:

N=([1 + r]/r)([Z_1–α/2_ + Z_1–β_]^2^/d^2^) + (Z^2^_1–α/2_)/2(1 + r)

where *Z*_1–α/2_ at the 5% level of significance = 1.96; *Z*_1–β_ at 80% power = 0.84; ratio allocation (group 2/group 1) = 1.

Sample size (n) = ([1 + 1]/1)([1.96 + 0.84]^2^/0.8^2^) + (1.96)^2^/2(1 + 1) = 26 per group.

Considering a 15% dropout (n=4), a total of 30 samples are required per group.

### Sample Size

The sample size calculation used the Cohen *d* formula to compare 2 means. The effect size (*d*) was estimated using the following equation:

Effect size = *d* = (*μ*_2_ – *μ*_1_)/*σ* = 0.8

Given a large effect size (*d*=0.8), the required sample size per group was determined using the following formula:

*N*=([1 + *r*]/*r*)([*Z*_1–α/2_ + *Z*_1–β_]^2^/*d*^2^) + (*Z*^2^_1–α/2_)/2(1 + *r*)

where *Z*_1–α/2_ at the 5% level of significance = 1.96; *Z*_1–β_ at 80% power = 0.84; ratio allocation (group 2/group 1) = 1.

Substituting these values:

n=([1 + 1]/1)([1.96 + 0.84]^2^/0.8^2^ + (1.96)^2^/2(1 + 1)

n=2×([2.8]^2^/[0.64])+(3.84/4)

n=2 × (7.84/0.64) + 0.96

n=2 × 12.25 + 0.96

n=24.5 + 0.96= 25.46

Rounding up, 26 participants per group are required. Considering a 15% dropout rate (approximately 4 participants per group), the final sample size will be 30 participants per group, totaling 60 participants for the study.

### Safety Outcomes

Adverse events will be reported as they occur. No negative effects are expected.

### Statistical Analysis

For demographic factors, all data will be summarized using baseline characteristics, expressed as the mean and SD for continuous data and as frequency and percentage for categorical data.

The analysis of outcome variables will primarily focus on continuous variables. For parametric data, the minimum, maximum, mean, SD, SE, and 95% CI will be summarized. The normality of continuous outcome variables will first be tested using the Kolmogorov-Smirnov test at a 5% level of significance (*P*≤.05). If the data are found to deviate from normality, a nonparametric test will be applied to determine significance. An independent *t* test will be conducted for group comparisons to assess significant differences at the 5% level (*P*≤.05). The intervention groups will receive (1) cognitive training supplementing traditional physical treatment and (2) traditional physical treatment.

The effect size at different assessment visits between the 2 groups will be calculated for the variables MMSE and Montreal Cognitive Assessment. For nonnormal data, descriptive statistics will include the mean, median, and lower and upper quartiles. The Mann-Whitney test will be applied to determine statistical significance when nonparametric methods are required.

Categorical variables will be summarized using frequency (n) and percentage. Chi-square analysis will be performed to evaluate efficacy across categorical variables. An intention-to-treat analysis will be applied to minimize attrition bias among participants, including those lost to follow-up or deceased due to adverse effects of chemotherapeutic drugs.

### Monitoring Methods

#### Data Monitoring

The Data Monitoring Committee of Ravi Nair Physiotherapy College will be responsible for overseeing and monitoring the study data.

#### Monitoring Harms

Every instance of an adverse event will be reported to the Ethics Committee and the clinician responsible for evaluating and managing both anticipated and unanticipated adverse events, as well as any other unforeseen consequences arising from the trial interventions or trial conduct.

## Results

As of November 9, 2024, we enrolled 60 participants. Data collection will occur from November 14, 2024, to November 14, 2025. We expect the results on the effects of cognitive rehabilitation on cognition and quality of life in patients with CICI to be significant, with the findings scheduled for publication on November 25, 2025.

## Discussion

### Anticipated Findings

This study will evaluate and compare the effects of cognitive rehabilitation and conventional therapy on cognition in patients with CICI. It is anticipated that this framework will significantly enhance the cognitive functions of patients receiving cognitive therapy as an intervention alongside conventional management protocols.

We hypothesize that cognitive training will result in measurable changes in brain magnetic resonance imaging, including improved perfusion, enhanced white matter connectivity (particularly involving the hippocampus), and increased brain volume (including the hippocampus). We have designed a randomized controlled trial (RCT) to investigate the effects of cognitive training and physical exercise on cognitive functioning in patients with cancer who have undergone chemotherapy and reported cognitive difficulties confirmed by standardized and validated neuropsychological assessments. Several underlying mechanisms may explain the effectiveness of the therapies included in this study. Cognitive rehabilitation and active exercises (such as weight training, aerobic training, dance, and yoga) may enhance physical capacity by counteracting reduced activity levels during or after cancer treatment [23]. Furthermore, increased physical activity can positively influence mental health. Psychosocial therapies promote activity management and stress coping, thereby reducing fatigue. Cognitive rehabilitation enhances neuroplasticity, enabling the brain to reorganize and adapt in response to injury, and helps develop compensatory strategies that allow individuals to manage cognitive deficits more effectively. It also contributes to building cognitive reserve, thereby reducing the risk of cognitive decline and promoting resilience. Several RCTs have demonstrated the efficacy of cognitive rehabilitation in improving cognitive function and quality of life, as well as in reducing symptoms of anxiety and depression in individuals with CICI [24]. Meta-analyses have further shown that cognitive rehabilitation is associated with significant improvements in cognitive function, particularly in attention and memory [25].

A systematic review examined the effects of interventions aimed at enhancing cognitive function in patients experiencing CRCI. Studies involving patients with metastatic cancer or preexisting cognitive deficits were excluded. The review included studies published between January 2011 and September 2022. Various interventions were identified for managing CRCI, and the outcome measures were found to be consistent across studies [26]. An RCT focusing on patients with cancer with CICIs also highlighted cognitive rehabilitation as an effective approach to address the cognitive deficits commonly observed following chemotherapy [18]. According to research, the majority of BC survivors, particularly those who have undergone chemotherapy, experience long-term cognitive abnormalities that significantly diminish their quality of life [27]. Executive functions—including working memory, cognitive flexibility, multitasking, planning, and attention—are among the most severely affected cognitive domains. Previous studies across various populations have shown that cognitive training, a behavioral approach used to address cognitive deficits, can lead to notable improvements in specific cognitive abilities such as executive functions. Kiesl et al [24] conducted an RCT to evaluate the effectiveness of cognitive training through a 12-week program and concluded that it resulted in significant improvements in processing speed, planning, and task performance. A study by Ferguson et al [28] evaluated the efficacy of cognitive behavioral therapy and memory and attention adaptation training (MAAT), both designed to improve cognitive function in patients with cancer. Following treatment, they concluded that cognitive behavioral therapy was more effective than MAAT; however, patients reported higher satisfaction with MAAT [29]. A systematic review by Peng et al [30] suggested that exercise interventions improve cognitive function and quality of life, as assessed through self-reported outcomes, and emphasized the need for higher-quality research to further explore exercise strategies in CRCI. Similarly, a systematic review by Hilfiker et al [20] assessed physiotherapeutic interventions for CRCI following cancer treatment and concluded that physiotherapy, including cognitive rehabilitation, was effective and led to significant improvements in cognition and quality of life.

An RCT investigated the feasibility and preliminary effectiveness of a novel online executive function training program for long-term BC survivors. Forty-one BC survivors (21 active and 20 waitlist) completed the 48-session training program over 12 weeks. The cognitive training resulted in significant improvements in cognitive flexibility, verbal fluency, and processing speed, with marginally significant downstream improvements in verbal memory as assessed using standardized measures [31]. According to Pendergrass et al [32], cognitive rehabilitation and training methods incorporating adaptive challenge levels, awareness practice, and repetitive skills training had a positive effect on cognitive function and self-reported quality of life. Similarly, a study by Wongarsa et al [33] on integrated interventions for mild cognitive impairment in older adults demonstrated that brain training activities—such as word guessing and memorization, image classification, music-based responses, basic arithmetic computation, and step-by-step recipe writing and cooking—greatly enhanced processing speed, attention, and memory. A systematic review by Melillo et al [34] encompassing studies conducted worldwide illustrated various approaches to managing cognitive impairment. Bellens et al [7] investigated the effects of web-based cognitive training, while in Canada (2018 and 2022), Campbell et al [35] and Duval et al [36] examined the impact of aerobic exercise on cognitive functions. Another study focused on the use of mindfulness-based stress reduction as an intervention [25].

### Strength

According to a consensus statement from the International Multidisciplinary Roundtable, cancer survivors can safely engage in sufficient exercise training to improve physical fitness, restore physical functioning, enhance quality of life, and reduce cancer-related fatigue. However, uncertainty remains regarding the effects of exercise on various outcomes, including peripheral neuropathy and cognitive functioning. The statement concluded that insufficient research exists on several aspects, such as anxiety, depressive symptoms, fatigue, health-related quality of life, cognitive decline, physical function, and the safety of exercise training for individuals who have or are at risk of developing BC-related lymphedema [35,37].

### Limitations

The majority of the available literature currently focuses on the most prevalent early stages of BC. Further research is needed to address a broader range of tumor types and to enhance the specificity of findings within the exercise oncology field. The limited number of trials directly comparing 2 or more levels of exercise training restricts understanding of dose-response relationships. Additionally, the lack of sufficient evidence regarding cognitive impairment outcomes highlights a clear knowledge gap.

Literature is scarce on the application of strategies and techniques for cognitive rehabilitation in managing CICI symptoms. Only a few studies have investigated interventions targeting memory, attention, information processing, and executive function in individuals with CICI. Moreover, there is no evidence regarding the role of physical therapy interventions and their outcomes in enhancing cognitive function. However, substantial evidence supports the positive effects of exercise on cognitive function in various clinical populations, including older adults. In cancer populations, where cognitive function is a primary outcome, further research is warranted. Cognitive function is commonly assessed using both self-reported measures and neuropsychological tests.

### Future Direction

The data on the prevalence rates of cognitive impairment in cancer survivors after chemotherapy accounted for variables such as age and time since therapy termination. Subgroup analyses and meta-regression were used to examine potential influences on prevalence estimates. The criteria for performing meta-regression were derived from Cochrane Guidance 35 [38]. A comprehensive meta-analysis was used to conduct the meta-regression. This review represents the first systematic comparison of prevalence rates of cognitive impairment in women with BC that has considered all methods used to ascertain impairment and evaluate long-term prevalence. Mean prevalence rates for CICI across all time points were 44% based on self-reports and 6% based on short cognitive screening [39]. The cognitive training intervention appears effective and may help reduce the deterioration of cognitive function in patients with CICIs.

### Conclusion

This RCT aims to provide a comprehensive rehabilitation strategy that simultaneously addresses physical and cognitive issues. Outcome measures such as the MMSE, Montreal Cognitive Assessment, Functional Assessment of Cancer Therapy-Cognitive, EORTC QLQ-C36, and the Patient-Specific Functional Scale will be used to evaluate the efficacy of the protocol. It is crucial to consider several challenges and limitations, including variability in patient responses, adherence to guidelines, and the need for skilled health care professionals to deliver the treatments consistently. Furthermore, a robust RCT with an adequate sample size and an appropriate control group is essential to establish the protocol’s effectiveness and generalizability in treating CICI.

## Appendix

Multimedia Appendix 1 SPIRIT checklist.

## Abbreviations

BC: breast cancer
CICI: chemotherapy-induced cognitive impairment
CONSORT: Consolidated Standards of Reporting Trials
CRCI: cancer-related cognitive impairment
EORTC QLQ-C30: European Organisation for Research and Treatment of Cancer Quality of Life Questionnaire
MAAT: memory and attention adaptation training
MMSE: Mini-Mental State Examination
RCT: randomized controlled trial
SPIRIT: Standard Protocol Items: Recommendations for Interventional Trials
